# Dr. George G. Jones

**Published:** 1904-03

**Authors:** 


Dr. George G. Jones died at his home at Geneseo, N. Y., January 25, 1904, aged 48 years. He graduated at the University of Buffalo in 1886.

